# Imaging and monitoring astrocytes in health and disease

**DOI:** 10.3389/fncel.2014.00074

**Published:** 2014-03-12

**Authors:** Carole Escartin, Keith K. Murai

**Affiliations:** ^1^CNRS CEA URA 2210 and CEA, DSV, I2BM, MIRCenFontenay-aux-Roses, France; ^2^Department of Neurology and Neurosurgery, Center for Research in NeuroscienceMontreal, Canada

**Keywords:** neuron-astrocyte interactions, reactive astrocytes, *in vivo* analysis, high-resolution imaging, brain imaging, electrophysiology, gene transfer, transgenesis

Astrocytes are key cellular partners to neurons in the brain. They play an important role in multiple processes such as neurotransmitter recycling, trophic support, antioxidant defence, ionic homeostasis, inflammatory modulation, neurovascular and neurometabolic coupling, neurogenesis, synapse formation, and synaptic plasticity. In addition to their crucial involvement in normal brain physiology, it is well known that astrocytes adopt a reactive phenotype under most acute and chronic pathological conditions such as ischemia, trauma, brain cancer, epilepsy, demyelinating, and neurodegenerative diseases. However, the functional impact of astrocyte reactivity is still unclear.

During the last decades, the development of innovative approaches to study astrocytes has significantly improved our understanding of their prominent role in brain function and their contribution to disease states. In particular, new genetic tools, molecular probes, and imaging techniques that achieve high spatial and temporal resolution have revealed new insight into astrocyte functions *in situ.*

This Research Topic illustrates how recent methodological advances have helped to uncover the role of astrocytes in health and disease. The articles assembled cover a range of approaches to both monitor astrocytes (high-resolution microscopy, live imaging, positron emission tomography, nuclear magnetic resonance, and electrophysiology) and manipulate their functional properties (optogenetics, mouse transgenesis, viral gene transfer, and human stem cell differentiation).

## Imaging and monitoring astrocytes

In their Technology report, Barros et al. (2013) discuss live imaging methods based on genetically-encoded optical biosensors to quantify, at the single-cell level and with high temporal resolution, the concentration, and dynamics of intracellular metabolites. In their Methods article, Perez-Alvarez et al. (2013) present a detailed methodological procedure to make the most of standard confocal microscopy and perform real-time imaging of astrocytes in the intact mouse brain. Barcia et al. (2013) further illustrate the potency of confocal microscopy to image, in fixed tissues, the microanatomy of astrocyte interactions with immune cells during neuroinflammatory processes. In their Methods article, Haseleu et al. (2013) describe an original technique to study another fine subcellular feature of astrocytes: the peripheral astrocyte process. This method is based on the analysis by conventional microscopy of acutely-dissociated astrocytes from the mouse brain. Dallérac et al. (2013) discuss how astrocytes are not silent in the brain and how studying astrocytes by electrophysiological recordings provides insight into their complex communication with neurons at the synapse. In their Original research article, Kabaso et al. (2013) use electrophysiology, this time combined with modeling, to describe the mechanical properties of vesicular release from astrocytes.

On the larger imaging scale, two articles present brain imaging techniques applied to the study of astrocytes. In his opinion article, Gurden (2013) discusses the evidence that astrocytes have a pivotal position to translate neuronal activity into hyperemic and blood oxygenation level dependent (BOLD) signals, which are measured by functional neuroimaging techniques. O'Brien et al. (2013) present several brain imaging methods to study astrocyte interactions with cerebral tumors *in situ*, including bioluminescence, fluorescent labeling of astrocytes, single photon emission computed tomography, positron emission tomography, and magnetic resonance imaging.

## Manipulating astrocytes

Li et al. (2013) provide a detailed review of new genetic and imaging tools to study neuron-astrocyte communication at the tripartite synapse. Central to this field, is the physiological manipulation of calcium levels in astrocytes and its precise monitoring with high spatial and temporal resolution. Davila et al. (2013) review the current molecular approaches to overexpress or down-regulate genes in astrocytes *in vivo* using mouse transgenesis or gene transfer. They illustrate the potency of these techniques to decipher astrocyte contribution to brain function. Merienne et al. (2013) describe the recently-developed viral vectors to achieve selective gene transfer in astrocytes *in situ*. These versatile tools can be used to model brain diseases involving astrocytes or to test astrocyte-based therapeutic strategies. Krencik and Ullian (2013) present the robustness and limits of using astrocytes derived from human pluripotent stem cells (hPSCs) to model or treat neurodevelopmental diseases. They provide a complete set of guidelines to optimize experiments with these cells.

## Multidisciplinary approaches to study the complex features of astrocytes

Finally, three Review articles extensively describe the multidisciplinary approach undertaken to understand some complex features of astrocytes. Stobart and Anderson (2013) describe our present knowledge on astrocyte contributions to neurometabolic and neurovascular coupling, and discuss how their dysfunction could participate to brain disorders. Through their historical review, Bouzier-Sore and Pellerin (2013) illustrate how the combination of biochemical analysis, live cellular imaging, magnetic resonance spectroscopy, transcriptomics, and metabolic modeling has contributed to the characterization of the unique metabolic features of astrocytes. Last but not least, Van Horn et al. (2013) provide a historical description of the discovery of D-serine, as a crucial gliotransmitter with multiple roles in brain development and function.

Overall, this Research Topic provides a collection of cutting-edge techniques, approaches, and models to study astrocytes in health and disease. It also suggests new directions to achieve discoveries on these fascinating cells.
